# 1-Hydr­oxy-3-(3-methyl­but-2-en­yloxy)xanthone

**DOI:** 10.1107/S1600536809040069

**Published:** 2009-10-13

**Authors:** Luis Gales, Raquel A. P. Castanheiro, Madalena M. M. Pinto, Ana M. Damas

**Affiliations:** a Instituto de Biologia Molecular e Celular, & Instituto de Ciências Biomédicas Abel Salazar, Portugal; b Centro de Química Medicinal da Universidade do Porto (CEQUIMED–UP), e Serviço de Química Orgânica, Faculdade de Farmácia, Universidade do Porto, Portugal

## Abstract

In the title compound, C_18_H_16_O_4_, a monoprenylated xanthone, the xanthone skeleton exhibits an essentially planar conformation (r.m.s. deviation 0.0072 Å) and the isoprenyl side chain remains approximately in the mean plane of the xanthone unit, making a dihedral angle of 4.5 (2)°. The hydroxyl group forms an intra­molecular O—H⋯O hydrogen bond. Moreover, there is a weak inter­molecular C—H⋯O inter­action between a ring C atom and the xanthene O atom. In the crystal structure, there are no inter­molecular hydrogen bonds and the crystallographic packing is governed by van der Waals forces, leading to an arrangement in which the mol­ecules assemble with their planes parallel to each other, having a separation of 3.6 (3) Å.

## Related literature

For a review of the biological activity of prenylated xanthones, see: Pinto *et al.* (2005 ▶). For background literature and synthesis of prenylated xanthones, see: Pinto *et al.* (2005 ▶); Epifano *et al.* (2007 ▶); Castanheiro *et al.* (2007 ▶). For the synthesis of the title compound using microwave radiation, see: Castanheiro *et al.* (2009 ▶). For analysis of related structures of xanthone derivatives, see: Gales *et al.* (2001 ▶, 2005 ▶ *a,b*); Castanheiro *et al.* (2007 ▶). For the interaction with biological membranes and target proteins, see: Maia *et al.* (2005 ▶); Epifano *et al.* (2007 ▶). For a review of prenylated xanthone crystal structures, see: Gales & Damas, 2005 ▶).
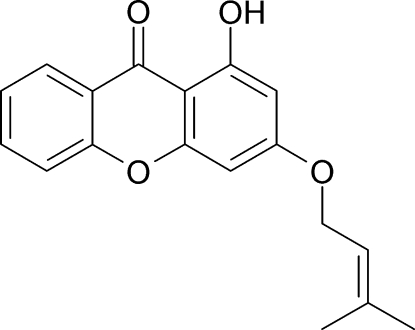


## Experimental

### 

#### Crystal data

C_18_H_16_O_4_
*M* *_r_* = 296.31
Triclinic, 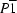

*a* = 4.8199 (3) Å
*b* = 11.7014 (8) Å
*c* = 13.6176 (10) Å
α = 77.329 (6)°
β = 88.582 (6)°
γ = 79.039 (6)°
*V* = 735.54 (9) Å^3^
*Z* = 2
Mo *K*α radiation
μ = 0.09 mm^−1^
*T* = 295 K
0.4 × 0.2 × 0.1 mm

#### Data collection

Oxford Diffraction Gemini PX Ultra CCD area-detector diffractometer
Absorption correction: none
8520 measured reflections
2981 independent reflections
1958 reflections with *I* > 2σ(*I*)
*R*_int_ = 0.017

#### Refinement

*R*[*F* ^2^ > 2σ(*F* ^2^)] = 0.048
*wR*(*F* ^2^) = 0.147
*S* = 1.07
2981 reflections
202 parameters
H-atom parameters constrained
Δρ_max_ = 0.16 e Å^−3^
Δρ_min_ = −0.15 e Å^−3^

### 

Data collection: *CrysAlis CCD* (Oxford Diffraction, 2004 ▶); cell refinement: *CrysAlis CCD*; data reduction: *CrysAlis RED* (Oxford Diffraction, 2004 ▶); program(s) used to solve structure: *SHELXS97* (Sheldrick, 2008 ▶); program(s) used to refine structure: *SHELXL97* (Sheldrick, 2008 ▶); molecular graphics: *ORTEP-3* (Johnson & Burnett, 1996 ▶); software used to prepare material for publication: *SHELXL97*.

## Supplementary Material

Crystal structure: contains datablocks I, global. DOI: 10.1107/S1600536809040069/bv2126sup1.cif

Structure factors: contains datablocks I. DOI: 10.1107/S1600536809040069/bv2126Isup2.hkl

Additional supplementary materials:  crystallographic information; 3D view; checkCIF report

## Figures and Tables

**Table 1:** Hydrogen-bond geometry (Å, °)

*D*—H⋯*A*	*D*—H	H⋯*A*	*D*⋯*A*	*D*—H⋯*A*
O1—H1*A*⋯O11	0.82	1.85	2.5846 (17)	148
C5—H5*A*⋯O2^i^	0.93	2.60	3.514 (2)	168

Symmetry code: (i) 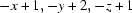.

**Fig. 1.**
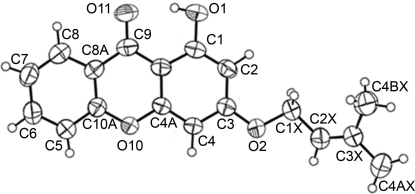
The molecular structure of the title compound, with atom labels and 50% probability displacement ellipsoids for non-H atoms.

**Fig. 2.**
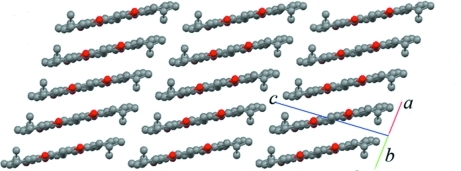
The packing of the title compound, showing parallel stacking planes 3.6Å apart. H atoms have been omitted.

## supplementary crystallographic information

### Comment

Prenylated xanthones have been reported to mediate a number of important biological activities, concerning a large variety of targets with therapeutic value. The presence of the prenyl side chains seems to enhance the interaction with biological membranes and with target proteins (Maia *et al.*, 2005 and Epifano *et al.*, 2007) and we plan to further study these kind of interactions.

However, the synthesis of prenylated xanthones usually involves toxic reagents and is considered not only very demanding but also environmentally unfriendly (Castanheiro *et al.*, 2007). We have looked for an alternative method to obtain prenylated xanthones. The title compound was the first example of a prenylated xanthone synthesized by the microwave irradiation method (Castanheiro *et al.*, 2009). In fact, microwave-assisted heating under controlled conditions is an invaluable technology for medicinal chemistry because it often dramatically reduces reaction times.

In the crystal, the title compound molecules are essentially planar (Fig. 1). The isoprenyl side chain adopts a nearly coplanar conformation relatively to the xanthone skeleton (corresponding dihedral angle 4.5 (2)°). This is an exception because in the crystal structures of other prenylated xanthones, the isoprenyl side chain is usually out of the plane of the xanthones moiety (for a review of prenylated xanthone crystal structures see: Gales & Damas, 2005). Moreover, the hydroxyl substituent bound to C1 forms a strong intramolecular hydrogen bond to O11 [O1—H1A···O11 = 2.5845 (17) Å].

In the crystal structure, the title compound forms stacking planes (Fig. 2) with intermolecular separation of 3.6 Å. The packing of the molecules is governed by van der Waals forces and there are no intermolecular hydrogen bonds.

### Experimental

Prenylation was carried out using prenyl bromide in alkaline medium under microwave irradiation according to the procedure reported by Castanheiro *et al.* (2009). Single crystals suitable for X-ray crystallographic analysis were grown by recrystallization from slow evaporation of a CH_2_Cl_2_/PE (60–80) solution.

### Refinement

Non-hydrogen atoms were refined anisotropically. The H atoms were positioned with idealized geometry using a riding model [O—H = 0.82, C—H = 0.93–0.97 Å]. All H atoms were refined with isotropic displacement parameters [set to 1.2 times of the *U*_eq_ of the parent atom (1.5 times for the methyl groups)].

### Crystal data

**Table:**

C_18_H_16_O_4_	*Z* = 2
*M**_r_* = 296.31	*F*(000) = 312
Triclinic, *P*1	*D*_x_ = 1.338 Mg m^−^^3^
Hall symbol: -P 1	Mo *K*α radiation, λ = 0.71073 Å
*a* = 4.8199 (3) Å	Cell parameters from 1141 reflections
*b* = 11.7014 (8) Å	θ = 4.0–24.3°
*c* = 13.6176 (10) Å	µ = 0.09 mm^−^^1^
α = 77.329 (6)°	*T* = 295 K
β = 88.582 (6)°	Plate, yellow
γ = 79.039 (6)°	0.4 × 0.2 × 0.1 mm
*V* = 735.54 (9) Å^3^	

### Data collection

**Table:**

Oxford Diffraction Gemini PX Ultra CCD area-detector diffractometer	1958 reflections with *I* > 2σ(*I*)
Radiation source: fine-focus sealed tube	*R*_int_ = 0.017
graphite	θ_max_ = 26.4°, θ_min_ = 2.6°
ω and θ scans	*h* = −5→6
8520 measured reflections	*k* = −14→14
2981 independent reflections	*l* = −17→16

### Refinement

**Table:**

Refinement on *F*^2^	Primary atom site location: structure-invariant direct methods
Least-squares matrix: full	Secondary atom site location: difference Fourier map
*R*[*F*^2^ > 2σ(*F*^2^)] = 0.048	Hydrogen site location: inferred from neighbouring sites
*wR*(*F*^2^) = 0.147	H-atom parameters constrained
*S* = 1.07	*w* = 1/[σ^2^(*F*_o_^2^) + (0.0771*P*)^2^ + 0.0559*P*] where *P* = (*F*_o_^2^ + 2*F*_c_^2^)/3
2981 reflections	(Δ/σ)_max_ < 0.001
202 parameters	Δρ_max_ = 0.16 e Å^−^^3^
0 restraints	Δρ_min_ = −0.15 e Å^−^^3^

### Special details

**Table:**

Geometry. All e.s.d.'s (except the e.s.d. in the dihedral angle between two l.s. planes) are estimated using the full covariance matrix. The cell e.s.d.'s are taken into account individually in the estimation of e.s.d.'s in distances, angles and torsion angles; correlations between e.s.d.'s in cell parameters are only used when they are defined by crystal symmetry. An approximate (isotropic) treatment of cell e.s.d.'s is used for estimating e.s.d.'s involving l.s. planes.
Refinement. Refinement of *F*^2^ against ALL reflections. The weighted *R*-factor *wR* and goodness of fit *S* are based on *F*^2^, conventional *R*-factors *R* are based on *F*, with *F* set to zero for negative *F*^2^. The threshold expression of *F*^2^ > σ(*F*^2^) is used only for calculating *R*-factors(gt) *etc*. and is not relevant to the choice of reflections for refinement. *R*-factors based on *F*^2^ are statistically about twice as large as those based on *F*, and *R*- factors based on ALL data will be even larger.

### Fractional atomic coordinates and isotropic or equivalent isotropic displacement parameters (Å^2^)

**Table:**

	*x*	*y*	*z*	*U*_iso_*/*U*_eq_	
O1	0.2991 (3)	0.54781 (11)	0.38058 (10)	0.0702 (4)	
H1A	0.4106	0.5456	0.3343	0.105*	
O2	0.0013 (3)	0.78593 (11)	0.61948 (9)	0.0652 (4)	
O10	0.6160 (2)	0.91544 (10)	0.36924 (8)	0.0558 (3)	
O11	0.6489 (3)	0.62252 (12)	0.24577 (10)	0.0734 (4)	
C1	0.3046 (3)	0.64516 (14)	0.41726 (13)	0.0525 (4)	
C2	0.1471 (3)	0.66156 (14)	0.50003 (12)	0.0538 (4)	
H2A	0.0408	0.6055	0.5307	0.065*	
C3	0.1479 (3)	0.76265 (15)	0.53766 (12)	0.0518 (4)	
C4	0.3038 (3)	0.84872 (15)	0.49224 (12)	0.0538 (4)	
H4A	0.3000	0.9171	0.5168	0.065*	
C4A	0.4623 (3)	0.82954 (14)	0.41054 (11)	0.0480 (4)	
C5	0.9287 (4)	0.99283 (16)	0.25015 (13)	0.0612 (5)	
H5A	0.9186	1.0567	0.2813	0.073*	
C6	1.0918 (4)	0.98563 (18)	0.16677 (14)	0.0689 (5)	
H6A	1.1906	1.0462	0.1408	0.083*	
C7	1.1124 (4)	0.89031 (18)	0.12043 (14)	0.0694 (5)	
H7A	1.2247	0.8870	0.0642	0.083*	
C8	0.9666 (4)	0.80095 (17)	0.15780 (13)	0.0634 (5)	
H8A	0.9809	0.7367	0.1269	0.076*	
C8A	0.7966 (3)	0.80542 (15)	0.24201 (12)	0.0520 (4)	
C9	0.6388 (3)	0.71111 (15)	0.28351 (13)	0.0546 (4)	
C9A	0.4708 (3)	0.72862 (14)	0.36983 (12)	0.0487 (4)	
C10A	0.7792 (3)	0.90220 (15)	0.28679 (12)	0.0516 (4)	
C1X	−0.1460 (4)	0.69521 (16)	0.67349 (13)	0.0652 (5)	
H1XA	−0.0128	0.6219	0.6991	0.078*	
H1XB	−0.2807	0.6790	0.6290	0.078*	
C2X	−0.2946 (4)	0.74034 (17)	0.75770 (14)	0.0716 (5)	
H2XA	−0.3558	0.8224	0.7476	0.086*	
C3X	−0.3486 (4)	0.67573 (16)	0.84547 (13)	0.0642 (5)	
C4AX	−0.5155 (5)	0.7288 (2)	0.92405 (18)	0.0985 (8)	
H4AA	−0.5531	0.8142	0.9028	0.148*	
H4AB	−0.4096	0.7055	0.9863	0.148*	
H4AC	−0.6910	0.7007	0.9334	0.148*	
C4BX	−0.2550 (6)	0.5424 (2)	0.87218 (17)	0.1053 (8)	
H4BA	−0.1111	0.5186	0.8268	0.158*	
H4BB	−0.4135	0.5052	0.8669	0.158*	
H4BC	−0.1808	0.5184	0.9399	0.158*	

### Atomic displacement parameters (Å^2^)

**Table:**

	*U*^11^	*U*^22^	*U*^33^	*U*^12^	*U*^13^	*U*^23^
O1	0.0865 (9)	0.0539 (7)	0.0832 (9)	−0.0265 (6)	0.0115 (7)	−0.0323 (6)
O2	0.0812 (8)	0.0638 (8)	0.0629 (7)	−0.0349 (6)	0.0260 (6)	−0.0248 (6)
O10	0.0642 (7)	0.0533 (7)	0.0592 (7)	−0.0242 (5)	0.0179 (5)	−0.0229 (5)
O11	0.0800 (8)	0.0676 (8)	0.0872 (9)	−0.0196 (7)	0.0159 (7)	−0.0447 (7)
C1	0.0567 (9)	0.0432 (9)	0.0611 (10)	−0.0120 (7)	−0.0055 (8)	−0.0158 (7)
C2	0.0588 (10)	0.0490 (10)	0.0579 (10)	−0.0207 (8)	0.0024 (8)	−0.0118 (8)
C3	0.0562 (9)	0.0508 (10)	0.0519 (9)	−0.0157 (7)	0.0032 (7)	−0.0145 (7)
C4	0.0635 (10)	0.0489 (9)	0.0579 (9)	−0.0211 (8)	0.0108 (8)	−0.0229 (8)
C4A	0.0511 (9)	0.0435 (9)	0.0530 (9)	−0.0138 (7)	0.0024 (7)	−0.0140 (7)
C5	0.0684 (11)	0.0575 (10)	0.0618 (10)	−0.0196 (9)	0.0132 (8)	−0.0161 (8)
C6	0.0727 (12)	0.0681 (12)	0.0644 (11)	−0.0201 (10)	0.0158 (9)	−0.0071 (9)
C7	0.0728 (12)	0.0791 (14)	0.0542 (10)	−0.0099 (10)	0.0166 (9)	−0.0157 (9)
C8	0.0668 (11)	0.0676 (12)	0.0573 (10)	−0.0061 (9)	0.0053 (9)	−0.0229 (9)
C8A	0.0504 (9)	0.0562 (10)	0.0503 (9)	−0.0056 (7)	0.0025 (7)	−0.0174 (8)
C9	0.0542 (9)	0.0522 (10)	0.0617 (10)	−0.0063 (7)	−0.0029 (8)	−0.0241 (8)
C9A	0.0476 (8)	0.0463 (9)	0.0545 (9)	−0.0088 (7)	−0.0027 (7)	−0.0160 (7)
C10A	0.0525 (9)	0.0540 (10)	0.0492 (9)	−0.0100 (7)	0.0050 (7)	−0.0140 (7)
C1X	0.0767 (12)	0.0563 (11)	0.0664 (11)	−0.0262 (9)	0.0178 (9)	−0.0117 (9)
C2X	0.0800 (13)	0.0569 (11)	0.0794 (13)	−0.0187 (9)	0.0279 (10)	−0.0161 (10)
C3X	0.0750 (12)	0.0612 (11)	0.0605 (10)	−0.0236 (9)	0.0129 (9)	−0.0141 (9)
C4AX	0.1244 (19)	0.0849 (16)	0.0881 (15)	−0.0254 (14)	0.0440 (14)	−0.0225 (13)
C4BX	0.163 (2)	0.0746 (15)	0.0736 (14)	−0.0225 (15)	0.0266 (15)	−0.0095 (11)

### Geometric parameters (Å, °)

**Table:**

O1—C1	1.3453 (19)	C7—C8	1.369 (3)
O1—H1A	0.8200	C7—H7A	0.9300
O2—C3	1.3548 (19)	C8—C8A	1.397 (2)
O2—C1X	1.4460 (18)	C8—H8A	0.9300
O10—C10A	1.3744 (19)	C8A—C10A	1.388 (2)
O10—C4A	1.3748 (18)	C8A—C9	1.463 (2)
O11—C9	1.247 (2)	C9—C9A	1.440 (2)
C1—C2	1.371 (2)	C1X—C2X	1.479 (3)
C1—C9A	1.417 (2)	C1X—H1XA	0.9700
C2—C3	1.389 (2)	C1X—H1XB	0.9700
C2—H2A	0.9300	C2X—C3X	1.316 (2)
C3—C4	1.398 (2)	C2X—H2XA	0.9300
C4—C4A	1.369 (2)	C3X—C4AX	1.495 (3)
C4—H4A	0.9300	C3X—C4BX	1.504 (3)
C4A—C9A	1.404 (2)	C4AX—H4AA	0.9600
C5—C6	1.374 (2)	C4AX—H4AB	0.9600
C5—C10A	1.391 (2)	C4AX—H4AC	0.9600
C5—H5A	0.9300	C4BX—H4BA	0.9600
C6—C7	1.384 (3)	C4BX—H4BB	0.9600
C6—H6A	0.9300	C4BX—H4BC	0.9600
			
C1—O1—H1A	109.5	O11—C9—C9A	122.80 (16)
C3—O2—C1X	117.02 (13)	O11—C9—C8A	121.86 (16)
C10A—O10—C4A	119.38 (13)	C9A—C9—C8A	115.34 (15)
O1—C1—C2	118.92 (15)	C4A—C9A—C1	116.83 (15)
O1—C1—C9A	119.70 (15)	C4A—C9A—C9	121.62 (14)
C2—C1—C9A	121.37 (15)	C1—C9A—C9	121.55 (15)
C1—C2—C3	119.42 (15)	O10—C10A—C8A	123.03 (15)
C1—C2—H2A	120.3	O10—C10A—C5	115.61 (15)
C3—C2—H2A	120.3	C8A—C10A—C5	121.36 (15)
O2—C3—C2	123.65 (14)	O2—C1X—C2X	107.68 (14)
O2—C3—C4	115.03 (14)	O2—C1X—H1XA	110.2
C2—C3—C4	121.31 (15)	C2X—C1X—H1XA	110.2
C4A—C4—C3	118.15 (15)	O2—C1X—H1XB	110.2
C4A—C4—H4A	120.9	C2X—C1X—H1XB	110.2
C3—C4—H4A	120.9	H1XA—C1X—H1XB	108.5
C4—C4A—O10	116.23 (14)	C3X—C2X—C1X	126.38 (18)
C4—C4A—C9A	122.89 (14)	C3X—C2X—H2XA	116.8
O10—C4A—C9A	120.88 (14)	C1X—C2X—H2XA	116.8
C6—C5—C10A	118.31 (18)	C2X—C3X—C4AX	122.55 (19)
C6—C5—H5A	120.8	C2X—C3X—C4BX	122.09 (19)
C10A—C5—H5A	120.8	C4AX—C3X—C4BX	115.33 (16)
C5—C6—C7	121.54 (18)	C3X—C4AX—H4AA	109.5
C5—C6—H6A	119.2	C3X—C4AX—H4AB	109.5
C7—C6—H6A	119.2	H4AA—C4AX—H4AB	109.5
C8—C7—C6	119.64 (17)	C3X—C4AX—H4AC	109.5
C8—C7—H7A	120.2	H4AA—C4AX—H4AC	109.5
C6—C7—H7A	120.2	H4AB—C4AX—H4AC	109.5
C7—C8—C8A	120.57 (17)	C3X—C4BX—H4BA	109.5
C7—C8—H8A	119.7	C3X—C4BX—H4BB	109.5
C8A—C8—H8A	119.7	H4BA—C4BX—H4BB	109.5
C10A—C8A—C8	118.56 (16)	C3X—C4BX—H4BC	109.5
C10A—C8A—C9	119.75 (15)	H4BA—C4BX—H4BC	109.5
C8—C8A—C9	121.69 (16)	H4BB—C4BX—H4BC	109.5
			
O1—C1—C2—C3	−179.05 (15)	C4—C4A—C9A—C9	179.75 (14)
C9A—C1—C2—C3	0.7 (2)	O10—C4A—C9A—C9	−0.2 (2)
C1X—O2—C3—C2	4.5 (2)	O1—C1—C9A—C4A	178.64 (14)
C1X—O2—C3—C4	−175.47 (14)	C2—C1—C9A—C4A	−1.1 (2)
C1—C2—C3—O2	−179.37 (14)	O1—C1—C9A—C9	−0.9 (2)
C1—C2—C3—C4	0.6 (2)	C2—C1—C9A—C9	179.34 (14)
O2—C3—C4—C4A	178.50 (13)	O11—C9—C9A—C4A	−179.71 (15)
C2—C3—C4—C4A	−1.5 (2)	C8A—C9—C9A—C4A	−0.2 (2)
C3—C4—C4A—O10	−178.95 (13)	O11—C9—C9A—C1	−0.2 (2)
C3—C4—C4A—C9A	1.1 (2)	C8A—C9—C9A—C1	179.34 (13)
C10A—O10—C4A—C4	−179.75 (12)	C4A—O10—C10A—C8A	0.2 (2)
C10A—O10—C4A—C9A	0.2 (2)	C4A—O10—C10A—C5	−179.70 (13)
C10A—C5—C6—C7	−1.0 (3)	C8—C8A—C10A—O10	179.40 (14)
C5—C6—C7—C8	0.3 (3)	C9—C8A—C10A—O10	−0.7 (2)
C6—C7—C8—C8A	0.2 (3)	C8—C8A—C10A—C5	−0.7 (2)
C7—C8—C8A—C10A	−0.1 (2)	C9—C8A—C10A—C5	179.25 (15)
C7—C8—C8A—C9	−179.96 (15)	C6—C5—C10A—O10	−178.90 (14)
C10A—C8A—C9—O11	−179.85 (15)	C6—C5—C10A—C8A	1.2 (3)
C8—C8A—C9—O11	0.1 (3)	C3—O2—C1X—C2X	−178.84 (14)
C10A—C8A—C9—C9A	0.6 (2)	O2—C1X—C2X—C3X	−149.12 (19)
C8—C8A—C9—C9A	−179.45 (13)	C1X—C2X—C3X—C4AX	−176.29 (19)
C4—C4A—C9A—C1	0.2 (2)	C1X—C2X—C3X—C4BX	1.5 (3)
O10—C4A—C9A—C1	−179.79 (13)		

### Hydrogen-bond geometry (Å, °)

**Table:**

*D*—H···*A*	*D*—H	H···*A*	*D*···*A*	*D*—H···*A*
O1—H1A···O11	0.82	1.85	2.5846 (17)	148
C5—H5A···O2^i^	0.93	2.60	3.514 (2)	168

Symmetry codes: (i) −*x*+1, −*y*+2, −*z*+1.
